# Trends, risk factors and outcomes of healthcare-associated infections in a neonatal intensive care unit in Italy during 2013–2017

**DOI:** 10.1186/s13052-020-0799-3

**Published:** 2020-03-18

**Authors:** Marina Silvia Scamardo, Pasquale Dolce, Eliana Pia Esposito, Francesco Raimondi, Maria Triassi, Raffaele Zarrilli

**Affiliations:** 1 Department of Public Health, University of Naples “Federico II”, Via S. Pansini n.5, 80131 Naples, Italy; 2 Division of Neonatology, Department of Medical Translational Sciences, University of Naples “Federico II”, Naples, Italy

**Keywords:** Neonatal intensive care unit, Healthcare-associated infections, Active surveillance, Birth weight, Device utilization

## Abstract

**Background:**

Healthcare-associated infections (HAIs) occur frequently in intensive care units (NICUs). The aim of this study was to analyze the results of surveillance of HAIs in a III level NICU in Naples, Italy during 2013–2017 and to compare with those obtained during 2006–2010.

**Methods:**

The surveillance included 1265 neonates of all birth weight (BW) classes with > 2 days NICU stay. Infections were defined using standard Centers for Disease Control and Prevention definitions adapted to neonatal pathology.

**Results:**

A total of 125 HAIs were registered during 2013–2017 with a frequency of 9.9% and an incidence density of 3.2 per 1000 patient days. HAIs occurred in all BW classes with a decreasing trend from the lowest to the highest BW classes (p = < 0.001). Central line-associated blood stream infection (CLABSI) was the most frequent infection (69.6%), followed by ventilator associated pneumonia (VAP) (20%), urinary tract infection (UTI) (8.8%) and necrotizing enterocolitis (NEC) (1.6%). Also, CLABSI and VAP incidence density decreased from lower to highest BW classes showing a significant trend (*p* = 0.007). Most frequent pathogens responsible for CLABSI were: Coagulase-negative staphylococci (CONS) (25.3%), *Candida parapsilosis* (21.8%), *Pseudomonas aeruginosa* (5.7), *Escherichia coli* and *Klebsiella pneumoniae* (6.8%). No microbiological diagnosis was achieved for 20.7% of CLABSI. *Pseudomonas aeruginosa* (28%), *Stenotrophomonas maltophilia* (20%), and CONS (20%) were the most frequent pathogens responsible for VAP. CLABSI incidence density showed no differences between 2006 and 2010 and 2013–2017, while VAP incidence density for the 751–100 g BW class was higher during 2006–2010 than during 2013–2017 (*p* = 0.006). A higher incidence of the CLABSI caused by Gram positive bacteria (*p* = 0.002) or by undetermined etiology (*p* = 0.01) was observed during 2013–2017 than during 2006–2010, while a significant lower incidence of VAP caused by Gram-negative bacteria was found during 2013–2017 than during 2006–2010 (*p* = 0.007).

**Conclusion:**

HAIs in the NICU developed in all BW classes with a decreasing trend from the lowest to the highest BW classes in both study periods. Differences in the aetiology of CLABSI and VAP were found between the two study periods. This reinforces the importance of HAIs surveillance protocol in the NICU, which monitors microbiological isolates and use of medical devices for all BW classes of neonates.

## Background

Healthcare-associated infections (HAIs) occur frequently in neonates admitted to intensive care units and recognizes many risk factors, including low birth weight and factors related to invasive procedures, such as vascular catheterization and mechanical ventilation [1, 2].

Active infection surveillance is recognized internationally as one of the activities that contribute to the reduction of the incidence of infections in hospitalized neonates [3–12]. Several active surveillance protocols for HAIs in neonatal intensive care units (NICUs) have been established worldwide, who differs in type of infections/outcomes and/or patients’ population included in the surveillance. In particular, the neonIN network in UK, the Vermont Oxford Network in USA and the Canadian Neonatal Network HAIs surveillance protocols focus mainly on early- and late-onset sepsis, while the NHSN and the NEO-KISS protocols extend surveillance of HAIs to VAPs and HAPs [10, 11]. Also, the majority of HAIs surveillance systems includes all patients’ population of NICUs [4–6, 8, 9, 12], while the NEO-KISS system monitors HAIs in infants of BW < 1500 g in NICUs until discharge, until death, or until they reach 1800 g [10, 11]. Moreover, benchmarking of HAIs surveillance protocols in NICUs is available on a national scale for NHSN [4–6], NEO-KISS [10, 11], the Vermont Oxford Network [7, 8], the neonIN network [12], and the Canadian Neonatal network [9]. A national framework for the surveillance of HAIs in Italy-SPIN-UTI project is active in Italy since 2008, which involves adult Intensive care units (ICUs) with none of NICUs [13, 14]. Although no surveillance network for HAIs in NICUs is present in Italy, few studies reported the prevalence [15], incidence of HAIs [16–20] and epidemics of specific nosocomial pathogens [20–24] in selected Italian NICUs.

We recently analyzed the results of HAIs surveillance in a III level NICU in Naples, Italy during 2006–2010 using the NHSN surveillance protocol [18]. Our data showed that HAIs developed in all BW classes, but low BW neonates were at major risk to acquire HAIs [18]. During the surveillance, we observed an outbreak of extensively drug-resistant (XDR) *Acinetobacter baumannii* in the NICU from November 2010 to July 2011, which required the temporary closure of the ward to external admissions of neonates during 2011 [21]. To better understand the risk factors of infection and the impact of prevention activities, we decided to expand our data and compare the results of surveillance of HAIs in the NICU using the NHSN surveillance protocol during 2013–2017 and to compare HAIs with those obtained during 2006–2010.

## Methods

### Setting

The NICU of the University Hospital of Naples “Federico II”, Italy is a III level Unit with a total of 25 incubators and cradles, 8 for intensive care and 17 for intermediate care. The ward serves the University Obstetric Clinic (approximately 2000 births/year) which is both a high-risk pregnancy center and an obstetric emergency service. Moreover, outborn neonates from the regional Newborn Emergency Transport Service are hospitalized in the NICU. In 2013–2017 years, a median (min, max) number of infants per year equal to 280 (246, 311) was admitted.

### Active surveillance

Healthcare-associated infections (HAIs) active patient-based surveillance (AS) is the identification of HAIs by trained personnel who proactively look for HAIs using multiple data sources. Data are collected from the medical records of the patients every week. The surveillance personnel conduct rounds on the ward to review the results from the laboratory and sometimes from the radiologic findings and to look on the medical records for signs and symptoms of infection, according to the standard definitions. Any clinical issues are directly discussed with caregivers. Data are analyzed on monthly basis and expressed as monthly report. Monthly report consists of patient data, data on swab isolations of sentinel pathogens, device utilization ratios and infection data. All neonates with > 2 days NICU stay enter the AS system and data regarding date of birth, birth weight (BW), gestational age, type of delivery, Apgar score, date of admission in the ward, date of infection, discharge date, type of microorganisms isolated, use of invasive devices (days of central line catheterization, including umbilical catheterization, and invasive ventilation), antimicrobial therapy exposure and infections are collected. The end of the surveillance period coincides with the discharge of the newborn from the ward. Infections are defined using standard Centers for Disease Control and Prevention (CDC) definitions adapted to neonatal pathology [3] and are considered to be health care associated if they develop > 2 days after NICU admission. VAP was defined using CDC criteria for defining nosocomial pneumonia for infants ≤ 1 year old [25]. We exclude neonates with congenital or perinatal infection. This paper analyzes data from the HAIs AS system over a 5 years period (2013–2017). For this study purposes, only CLABSI, pneumonia, necrotizing enterocolitis (NEC), and urinary tract infections (UTIs) were considered. Central line-associated blood-stream infections (CLABSI) and ventilator-associated pneumonia (VAP) were attributed if a central line, including umbilical, catheter and invasive ventilation, respectively, were in place at the time of or within 48 h prior to the development of the infection. The etiology of all infections within each BW class was assessed during the study period. HAIs surveillance was regulated by the Regional Health Authority and it was one of the basic components of the Regional Plan for Healthcare-associated infections Prevention and Control [26]. Results of surveillance activities were periodically reported to the Regional Health Authority.

### Statistical analysis

Descriptive data of device-associated infections were expressed as absolute number, percentage and incidence densities (presented per 1000 specific device-days) with respective 95% confidence intervals (CIs). If a neonate had multiple episodes of HAIs, each episode was considered as an independent event. The total number of device-associated infections and the total days of device utilization were computed for each birth weight class. Frequency measures were calculated as percent of infection and as incidence densities, i.e. infection rates per 1000 patient days or 1000 days of directly related invasive device within 5 BW categories (≤750 g,751–1000 g, 1001–1500 g, 1501–2500 g, and ≥ 2501 g). Device utilization rates within such classes were also calculated. To detect whether a significant birth weight trend occurred, we fitted a generalized linear model with a Poisson link function. The log transformed total device-days value was used as an offset in the model. Separate models were fitted for device-associated CLABSI, and VAPs.

To compare the second study period with those obtained during 2006–2010 study period, we included in the models an interaction terms (birth weight*study period) to explore whether there were differences in HAI density between the two study periods by birth weight class.

Generalized linear model with a Poisson link function was performed also to test for differences among etiology of device-associated infections, using gram negatives as reference group. To compare the two study periods also in terms of etiology of device-associated infections, the interaction terms (etiology *study period) was included in the models. Separate models were fitted for CLABSI and VAPs. All statistical analyses were performed using the R software environment for statistical computing, version 3.6.0 [27]. For all statistical analysis, a *p*-value< 0.05 was considered as statistical significance.

## Results

During 2013–2017, 1265 neonates, corresponding to 90.68% of all admissions to the ward, entered the HAIs AS system with a total of 39,207 days of stay, 12,140 days of use of central-line catheter and 7591 days of mechanical ventilation (Table 1). The remaining 9.32% of admissions was excluded because either ineligible (length of stay < 2 days) or missed by the HAIs AS system. During the study period, neonates with > 1000 g BW accounted for 65.86% of total patient days (1001–1500 g, 1501–2500 g, and ≥ 2501 g classes representing 23.6, 28.7, and 13.6%, respectively). Neonates with extremely low BW (ELBW) (≤750 g and 751–1000 g BW classes) were 34.14% of patient days in the NICU accounting for 16.1 and 18.04% of total patient days, respectively).

**Table 1:** Neonates data per birthweight category during the study period

Year	2013	2014	2015	2016	2017	Total
Hospitalized patients	246	274	311	280	284	1395
Surveilled patients	232	248	287	254	244	1265
**Number of patient days**^**a**^	**8186**	**7598**	**7707**	**7604**	**8112**	**39,207**
≤ 750 g	1583	990	1083	1502	1156	6314
751–1000 g	1753	2064	1328	820	1108	7073
1001–1500 g	1654	1666	1557	1897	2476	9250
1501–2500 g	2043	2035	2566	2385	2222	11,251
> 2500 g	1153	843	1173	1000	1150	5319
**Number of central line days**^**a**^	**2442**	**2190**	**2549**	**2479**	**2480**	**12,140**
≤ 750 g	577	477	519	695	405	2673
751–1000 g	629	725	551	292	336	2533
1001–1500 g	503	422	557	620	929	3031
1501–2500 g	490	383	637	704	610	2824
> 2500 g	243	183	285	168	200	1079
**Number of ventilator days**^**a**^	**2018**	**932**	**1141**	**1774**	**1726**	**7591**
≤ 750 g	897	323	407	826	506	2959
751–1000 g	529	371	406	220	341	1867
1001–1500 g	228	89	134	300	523	1274
1501–2500 g	280	75	90	363	294	1102
> 2500 g	84	74	104	65	62	389

^a^Data refer to cases per 1000 specific device-days

**Fig. 1:**
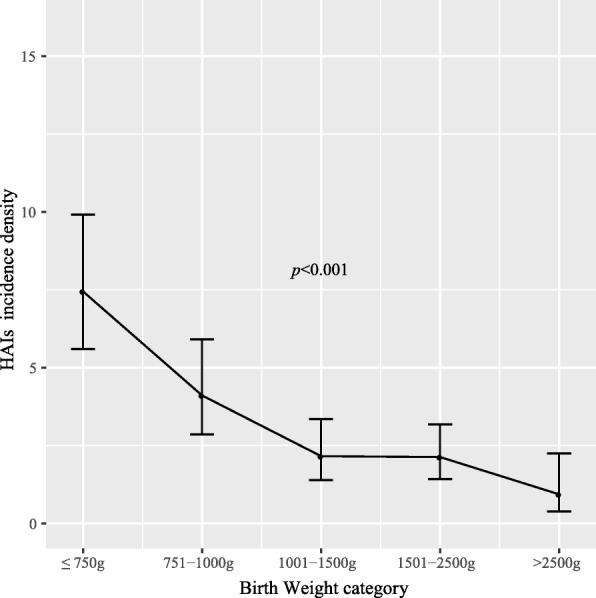
Trend of HAIs incidence densities (95% CI) per 1000 patient days across BW categories. *P*-values are obtained from Poisson Regression for testing whether incidence densities significantly vary across BW categories

**Fig. 2:**
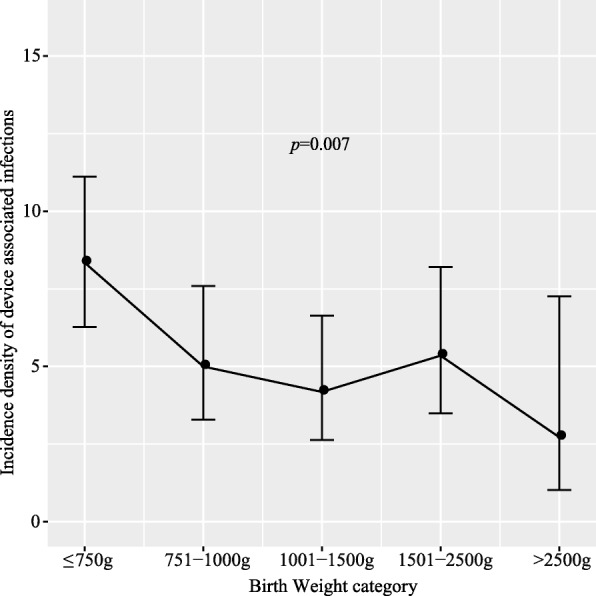
Trend of incidence densities (95% CI) of device-associated infections per 1000 days of device utilization across BW categories. *P*-values are obtained using Poisson Regression for testing whether incidence densities significantly vary across BW categories

The overall number of HAIs registered during the study period by the local AS system was 125 (which corresponded to a total infection rate of 9.9% and a total incidence density of 3.2 per 1000 patient days). The crude mortality rate of the patients under surveillance was 5.45%. The mortality rate of the infected patients was 19.4%. HAIs developed in all BW classes, but 60.8% of all HAIs developed in patients of ≤1000 g weight at birth. Also, a significant decreasing trend of incidence density of HAIs per 1000 patient days was observed from the lowest to the highest BW classes (7.44, 4.1, 2.16, 2.13 and 0.94 in ≤750 g, 751–1000 g, 1001–1500 g, 1501–2500 g, and ≥ 2501 g BW newborns, respectively, *p* < 0.001) (Fig. 1). CLABSIs proved to be the most frequent infections (69.6%), followed by VAPs (20%), UTIs (8.8%), and necrotizing enterocolitis (NECs) (1.6%). Device associated infections (i.e. CLABSIs and VAPs) represented 89.6% of HAIs. A significant decreasing trend from the lowest to the highest BW classes (*p* = 0.007) was found for incidence densities of device associated infections during 2013–2017 study period (Fig. 2). Incidence densities of CLABSIs and VAPs across the five BW classes are shown in Table 2. The incidence density of CLABSIs decreased significantly from the lowest to the highest BW classes (*p* = 0.001), while the incidence density of the VAPs decreased in the first three classes of BW and increased in the 1501–2500 g and ≥ 2501 g BW classes, showing a non-significant trend (*p* = 0.174).

**Table 2:** Incidence densities of device-associated infections per birth weight category in surveilled neonates

Device-associated infection	≤ 750 g	751–1000 g	1001–1500 g	1501–2500 g	> 2500 g	***p***-value
CLABSI	11,972	7106	5609	6020	2780	0.001
VAP	5069	2142	0,785	3630	2571	0.174

*Abbreviations*: *CLABSI* central line-associated bloodstream infection, *VAP* ventilator-associated pneumonia

Aetiology of device-associated infections among the five BW classes is shown in Table 3. Most frequent pathogens responsible for CLABSIs were: Coagulase-negative staphylococci (CONS) (25.3%), *Candida parapsilosis* (21.8%), *Candida albicans* (4.6%), *Pseudomonas aeruginosa* (5.7%), *Escherichia coli* and *Klebsiella pneumoniae* (6.8%). No microbiological diagnosis was achieved for 20.7% of CLABSIs. *P. aeruginosa* (28%), *Stenotrophomonas maltophilia* (20%), and CONS (20%) were the most frequent pathogens responsible for VAP. During the study period, the incidence density of Gram-positive CLABSI was significantly higher than the incidence density of Gram-negative CLABSI (2.47 and 1.40, respectively) (*p* = 0.008). On the other hand, the incidence density of VAPs caused by Gram negative was significantly higher than that caused by Gram-positive (2.11 and 0.26, respectively) (*p* < 0.001) and by polymicrobial aetiology (2.11 and 0.92, respectively) (*p* = 0.001). No microbiological diagnosis was achieved in 16% of infections.

**Table 3:** Etiology of device-associated infections per birth weight category in surveilled neonates

CLABSI	≤750 g	751–1000 g	1001–1500 g	1501–2500 g	> 2500 g	Total (%)
CONS	8	4	5	4	1	**22 (25.3)**
*Candida parapsilosis*	7	3	6	2	1	**19 (21.8)**
Not determined	8	3	3	4		**18(20.7)**
*Pseudomonas aeruginosa*	2	2	1			**5 (5.7)**
*Candida albicans*	1	2		1		**4 (4.6)**
*Escherichia coli*	1	1		1		**3 (3.4)**
*Escherichia coli* ESBL+	2			1		**3 (3.4)**
*Klebsiella pneumoniae*	2			1		**3 (3.4)**
*Klebsiella pneumoniae* ESBL+		2	1			**3 (3.4)**
*Staphylococcus aureus*	1			1		**2 (2.3)**
*Enterococcus faecalis*				2		**2 (2.3)**
*Streptococcus sanguinis*					1	**1 (1.1)**
*Kocuria kristinae*		1				**1 (1.1)**
*Candida pelliculosa*			1			**1 (1.1)**
**Total (% within BW category)**	**32 (36.8)**	**18 (20.7)**	**17 (19.5)**	**17 (19.5)**	**3 (3.4)**	**87 (100)**
**VAP**	**≤ 750 g**	**751–1000 g**	**1001–1500 g**	**1501–2500 g**	**> 2500 g**	**Total (%)**
*Pseudomonas aeruginosa*	4	1		2		**7 (28)**
CONS	4	1				**5 (20)**
*Stenotrophomonas maltophilia*	4	1				**5 (20)**
*Staphylococcus aureus*	1	1				**2 (8)**
*Acinetobacter baumannii*	1			1		**2 (8)**
*Enterobacter cloacae*			1			**1 (4)**
*Klebsiella pneumoniae* ESBL+	1					**1 (4)**
Polymicrobial (*Pseudomonas aeruginosa + Candida parapsilosis*)				1		**1 (4)**
Polymicrobial (*Pseudomonas aeruginosa + Klebsiella pneumoniae* ESBL+)					1	**1 (4)**
**Total (% within BW category)**	**15 (60)**	**4 (16)**	**1 (4)**	**4 (16)**	**1 (4)**	**25 (100)**

The most frequent device-unrelated HAIs were UTIs (8.8% of all), which mainly affected neonates belonging to the 751–1000 g and 1501–2500 g BW classes (distribution of UTIs was 0, 54.5, 9.1, 27.3 and 9.1% in ≤750 g, 751–1000 g, 1001–1500 g, 1501–2500 g, and ≥ 2501 g BW newborns, respectively). Fifty-five percent of UTIs were caused by *E. coli* (18.18%), *K. pneumoniae* (18.18%) and *Enterobacter spp.* (18.18%), the remaining 45.5% were caused by *Klebsiella oxytoca*, *Enterococcus faecalis* and polymicrobial aetiologies. In the 2013–2017 study period, we had two cases of Necrotizing Enterocolitis (NEC) and no aetiology was defined in both of cases.

Then, a comparison was performed between 2013 and 2017 study period and 2006–2010 study period [18]. The number of patient days was 43,447 in the first period and 39,207 in the second period, the number of central line utilization days was 4232 in the first period and 12,140 in the second period and the number of ventilation utilization days was 5208 in the first period and 7591 in the second period. Table 4 shows the distribution of patient days and devices utilization across BW classes during the two study periods. The number of patient days, central line utilization and ventilation utilization increased by 1.5-fold in the ≤750 g BW class during 2013–2017 study period, while percentages of devices utilization decreased in the > 2500 g BW class during 2013–2017 study period. No other relevant differences were found between the two study periods (Table 4).

**Table 4:** Percentages of patient days and devices utilization across BW classes during the two study periods

	≤ 750 g	751–1000 g	1001–1500 g	1501–2500 g	> 2500 g
**Patient days**					
2006–2010	9%	17%	27%	28%	19%
2013–2017	16%	18%	24%	29%	13%
**Central line utilization**					
2006–2010	13%	21%	29%	22%	15%
2013–2017	22%	21%	25%	23%	9%
**ventilation utilization**					
2006–2010	26%	31%	18%	15%	10%
2013–2017	39%	25%	17%	14%	5%

Incidence density of device associated infections per 1000 days of catheter or ventilation utilization during 2013–2017 were then compared to the incidence density obtained during 2006–2010 [18]. No significant differences were found between the two periods (6.17 for the first period vs. 5.67 for the second period, *p* = 0.548). Furthermore, no significant differences between the two periods were found when CLABSI incidence densities per 1000 catheter days were compared between the two periods (5.58 for the first period vs. 7.16 for the second period, *p* = 0.206). In contrast, VAP incidence densities per 1000 days of ventilation utilization were significantly different between the two periods (6.91 for the first period vs. 3.29 for the second period, *p* = 0.004).

**Fig. 3:**
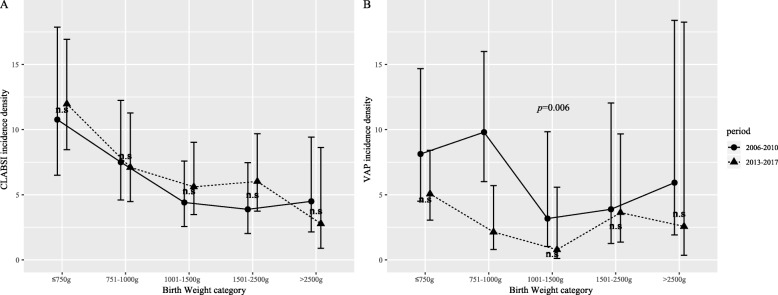
Comparison between the two study periods (2006–2010 and 2013–2017) for CLABSI incidence densities (95% CI) per 1000 catheter days across BW categories (**a**) and VAP incidence densities (95% CI) per 1000 days of ventilation utilization across BW categories (**b**). *P*-values are obtained using Poisson Regression for testing whether incidence densities significantly differ between the two period for each BW category

**Fig. 4:**
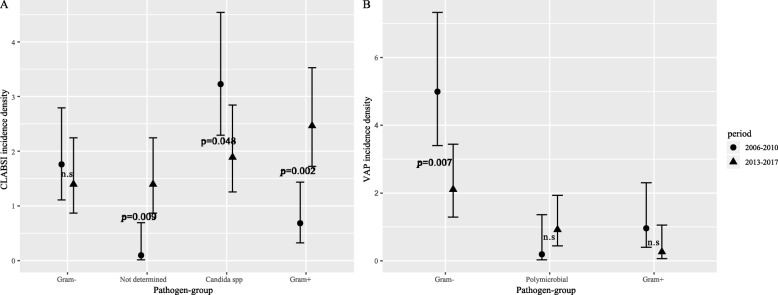
Comparison between the two study periods (2006–2010 and 2013–2017) for the CLABSI incidence densities (95% CI) per 1000 catheter days by etiology (**a**) and VAP incidence densities (95% CI) per 1000 days of ventilation utilization by etiology (**b**). *P*-values are obtained using Poisson Regression for testing whether incidence densities significantly differ between the two periods for each etiology

Finally, the incidence densities of CLABSI per 1000 catheter days by birth weight class and incidence densities of VAPs per 1000 days of ventilation utilization by birth weight class during 2013–2017 were compared to device-associated infection incidence densities during 2006–2010. There were no significant differences between the two periods for CLABSIs, whose incidence densities showed a decreasing trend from the lowest to the highest BW classes in both study periods (Fig. 3a). On the other hand, the incidence of VAPs in the 751–1000 g BW class was significant higher during 2006–2010 than 2013–2017 study period (*p* = 0.006) (Fig. 3b). Moreover, a significant higher incidence density of CLABSIs caused by Gram-positive bacteria (*p* = 0.002) and by undetermined aetiologies (*p* = 0.009), and a significant lower incidence of VAPs caused by Gram-negative bacteria (*p* = 0.007) was found during 2013–2017 compared to the 2006–2010 study period (Fig. 4a and b, respectively). The crude mortality rate of the patients under surveillance in the 2006–2010 study period was 4.83%. The mortality rate of the infected patients was 14.9%. Both the crude mortality rate and the mortality rate of infected patients increased during 2013–2017 compared with the 2006–2010 study period, being 5.45% vs 4.83 and 19.4% vs 14.9%, respectively.

## Discussion

HAIs are frequent complications occurring during hospitalization of newborns in NICUs and are associated with patients’ susceptibility conditions such as prematurity and immune status, use of invasive devices, such as central and/or umbilical vascular catheterization and mechanical ventilation, total parenteral nutrition, antimicrobial use and other concomitant drugs therapeutic variables [1, 2]. We recently reported the results of the surveillance of HAIs in a NICU in Italy using the NHSN surveillance protocol [18].

In the present study, we analyzed the results of surveillance of HAIs during 2013–2017 and compared them with HAIs during 2006–2010 in the same NICU [18] as internal benchmarking. During 2013–2017, total infection rate and total incidence density per 1000 patient days of HAIs were similar to those found during 2006–2010 (9.9% and 3.2 vs. 9% and 3.5). In partial accordance with our data, a single center cohort study in an Italian NICU reported an infection rate of 13.2% and an incidence density of 7.8 HAIs per 1000 patient days [16] and a multicenter prospective cohort study in six Italian NICUs reported an infection rate of 12.8% and an incidence density of 6.93 HAIs per 1000 patient-days [17]. Also, a multicenter retrospective cohort study in pediatric intensive care units (PICUs) and NICUs in Italy and Brazil during 2010–2014 described a cumulative incidence of HAI of 3.6/100 ICU admissions and an infection rate of 3.6/1000 ICU days [19].

Similarly to HAIs occurred during 2006–2010 study period in the same NICU [18], HAIs were more frequent in low birth weight groups (< 1000 g 60.8%; in detail BW1 < 750 g 37.6% and BW2 750-1000 g 23.20%) but developed in all BW classes. This reinforces the need to surveil all BW classes in NICUs according to NHSN protocol [3–6] and not infants of BW ≤ 1500 g according to NEO-KISS protocol [10, 11].

During 2013–2017 study period, device associated infections, i.e. CLABSIs (69.6%) and VAPs (20%), represented 89.6% of HAIs in our NICU. Incidence density of device associated infections did not significantly change between the two study periods (6.17 for the first period vs. 5.67 for the second period, *p* = 0.548). An increase of CLABSI incidence densities per 1000 catheter days, although not significant, was observed during 2013–2017 period respect to 2006–2010 period (7.16 versus 5.58 *p* = 0.206), which can be dependent by the increase in the number of patient days and central line utilization in the ≤750 g BW class in the second study period (Table 4). This finding is in agreement with previous studies showing that bloodstream infections prevailed among HAIs in Italian NICUs [16, 17] and that bloodstream infections were the main infections (45,4%), followed by lower respiratory tract infections (27.8%) and urinary tract infections (15.8%) in Italian and Brazilian PICUs and NICUs [19]. High percentage of bloodstream infections in NICUs has been also reported by NHSN in USA [4], neonINnetwork in UK [12] and worldwide [28–31]. Finally, the increase of CLABSI incidence density during 2013–2017 in our NICU is alarming because CLABSI rates measure Hospital performance for high-quality patient care [32].

On the other hand, a significant increase of VAP incidence density in our NICU was found for the 751–1000 g BW class during 2006–2010 compared with 2013–2017 period (Fig. 3b). This might have been dependent on the increase in VAPs in very-low birth weight neonates caused by two outbreaks during 2006–2010 period in the NICU [21, 24]. The elevated number of device-associated infections in the NICU strengthen the importance to calculate device utilization rates and use as risk factors for the development of CLABSIs and VAPs according to NHSN surveillance protocol [3–5]. Also, in agreement with previous surveillance studies of HAIs in other NICUs [16, 19, 29] and in the same NICU during 2006–2010 [18], UTIs were the third most frequent cause of HAIs after CLABSIs and VAPs in our NICU, but decreased from 28.8% [18] to 8.8% during 2013–2017.

Both the crude mortality rate and the mortality rate of infected patients increased during 2013–2017 compared with the 2006–2010 study period. This could have been contributed by the higher number of patient days, central line utilization and ventilation utilization in the ≤750 g BW class and the higher number of CLABSI during 2013–2017 compared with 2006–2010 study period.

Additional epidemiological information was provided by the analysis of the incidence densities of etiology of CLABSIs and VAPs in our NICU during 2013–2017 and the comparison with those found during 2006–2010. During 2013–2017, Gram-positive bacteria, in particular CONS (25.3%) and *S. aureus* (2.3%), were the most frequent pathogens responsible for CLABSIs. This finding is in accordance with several reports showing that gram-positive bacteria are the main cause of bloodstream infections in neonates in the NICUs [4, 12, 17, 28–30]. Other most frequent pathogens responsible for CLABSIs in the NICU were *Candida spp*., *C. parapsilosis* (21.8%) and *C. albicans* (4.6%), *P. aeruginosa* (5.7%), *E. coli* (3.4%) and *K. pneumoniae* (3.4%). During 2013–2017, VAPs were most frequently caused by *P. aeruginosa* (28%), *S. maltophilia* (20%), and CONS (20%). UTIs represented the most frequent device-unrelated infection in the NICU during 2013–2017 and were most frequently caused by *E. coli* (18.18%), *K. pneumoniae* (18.18%) and *Enterobacter spp.* (18.18%). The comparison of the etiologies of CLABSIs in the NICU between the two study periods showed a significant higher incidence density of CLABSIs caused by Gram-positive bacteria (*p* = 0.002) and by undetermined aetiology (*p* = 0.01) and a significant lower incidence of CLABSIs caused by *Candida spp*. (*p* = 0.05) during 2013–2017 compared to 2006–2010 period. Moreover, a significant lower incidence of VAPs caused by Gram-negative bacteria (*p* = 0.007) were found during 2013–2017 compared to 2006–2010 period. The differences in the aetiologies of VAPs and CLABSIs between the two periods could have been due to the occurrence of *P. aeruginosa* and *A. baumannii* outbreaks in the NICU during the first study period [21, 24]. Moreover, an increase in bloodstream infections caused by *C. parapsilosis* was observed during 2009–2012 in the NICU [22], which might have been responsible for the high incidence of CLABSIs caused by *Candida spp*. The increase of CLABSIs by undetermined aetiology during 2013–2017 it is worrying and without a definite cause.

We recognize that our study has limitations that affect the generalization of our results. The first limitation relies on the retrospective nature of the study, which did not allow to evaluate the efficacy of specific infection control measures to prevent HAIs in the NICU. One other limitation relies on the single center nature of the study, which did not allow to compare results among different clinical settings and to create a benchmarking on a national scale. Additional limitation of the study was the lack of analysis of inborn and outborn status, total parenteral nutrition, antimicrobial use and other concomitant drugs therapeutic variables of neonates included in the study. Future studies will be necessary to investigate the above issues.

## Conclusion

HAIs in our NICU during 2013–2017 developed in all BW classes with a decreasing trend from the lowest to the highest BW classes. CLABSIs, VAPs and UTIs were the most frequent HAIs. The use of central line catheter and mechanical ventilation invasive devices was associated with high risk of HAIs in our NICU. An increase of CLABSI incidence densities per 1000 catheter days, although not significant, was observed during 2013–2017 period respect to 2006–2010 period. Also, an higher incidence of the CLABSIs caused by Gram-positive bacteria or by undetermined etiology and a lower incidence of VAPs caused by Gram-negative bacteria were found during 2013–2017 study period respect to 2006–2010 period. This reinforces the importance of device associated HAIs surveillance protocol in the NICU, which monitors microbiological isolates responsible for infection and use of central line and assisted ventilation in all BW classes of neonates.

## Data Availability

The dataset supporting the conclusions of this article will be made available from the corresponding author upon request.

## Abbreviations

AS: Active surveillance
AV: Assisted ventilation
BSI: Bloodstream infection
BW: Birth weight
CLABSI: Central line-associated bloodstream infection
CVC: Central venous catheter
ELBW: Extremely low birth weight
HAIs: Healthcare-associated infections
NICU: Neonatal intensive care
VAP: Ventilator associated pneumonia
